# Glycogen Synthase Kinase 3β in Cancer Biology and Treatment

**DOI:** 10.3390/cells9061388

**Published:** 2020-06-03

**Authors:** Takahiro Domoto, Masahiro Uehara, Dilireba Bolidong, Toshinari Minamoto

**Affiliations:** Division of Translational and Clinical Oncology, Cancer Research Institute, Kanazawa University, Kanazawa 920-0934, Japan; tdomoto@staff.kanazawa-u.ac.jp (T.D.); masahiro.uehara@gmail.com (M.U.); dilireba929@stu.kanazawa-u.ac.jp (D.B.)

**Keywords:** glycogen synthase kinase 3β, cancer, biology, treatment

## Abstract

Glycogen synthase kinase (GSK)3β is a multifunctional serine/threonine protein kinase with more than 100 substrates and interacting molecules. GSK3β is normally active in cells and negative regulation of GSK3β activity via phosphorylation of its serine 9 residue is required for most normal cells to maintain homeostasis. Aberrant expression and activity of GSK3β contributes to the pathogenesis and progression of common recalcitrant diseases such as glucose intolerance, neurodegenerative disorders and cancer. Despite recognized roles against several proto-oncoproteins and mediators of the epithelial–mesenchymal transition, deregulated GSK3β also participates in tumor cell survival, evasion of apoptosis, proliferation and invasion, as well as sustaining cancer stemness and inducing therapy resistance. A therapeutic effect from GSK3β inhibition has been demonstrated in 25 different cancer types. Moreover, there is increasing evidence that GSK3β inhibition protects normal cells and tissues from the harmful effects associated with conventional cancer therapies. Here, we review the evidence supporting aberrant GSK3β as a hallmark property of cancer and highlight the beneficial effects of GSK3β inhibition on normal cells and tissues during cancer therapy. The biological rationale for targeting GSK3β in the treatment of cancer is also discussed at length.

## 1. GSK3β Biology in Normal Cells and Disease

Glycogen synthase kinase (GSK)3β is an isoform of the GSK3 family of kinases. It regulates many fundamental biological processes in cells by phosphorylating serine and threonine residues and thus interacting with more than 100 functional and structural proteins [1,2,3,4]. The enzymatic activity of GSK3β is finely tuned through differential phosphorylation of its serine (S)9 (inactive form) and tyrosine (Y)216 (active form) residues. GSK3β is normally active in cells, but negative regulation of its activity via S9 phosphorylation allows normal cells to maintain vital activities and homeostasis upon intra- and extracellular stimuli [3,4]. Deregulated expression and activity of GSK3β and/or impairment of its negative regulation contributes to the pathogenesis and progression of common diseases including type 2 diabetes mellitus, neurodegenerative disorders associated with cognitive deficit, chronic inflammatory and immunological diseases and cancer [5,6,7,8]. These functions in normal cells and in primary pathologies have highlighted GSK3β as a potential drug target in a broad spectrum of diseases, thereby expediting the rapid development of pharmacological GSK3β inhibitors [9,10,11].

The biochemistry, function and regulation of GSK3 family kinases (GSK3α, GSK3β) in normal cells and in disease have generated significant attention from chemistry, biomedical and pharmacology researchers. These have been extensively covered previously [3,4,5,6,7,8,9,10,11] and hence will not be the focus of this review. Instead, this review will focus on the critical roles that GSK3β has in cancer biology and treatment.

## 2. Overview of GSK3β Biology in Cancer

GSK3β is a crucial member of the Wnt/β-catenin-, hedgehog (Hh)-, notch- and c-myc-mediated major pro-oncogenic pathways, while also being a negative regulator of epithelial–mesenchymal transition (EMT) (reviewed in [12]). GSK3β has long been recognized to suppress tumor development and progression [12,13,14]. Several previous studies showed that GSK3β was inactivated mostly by S9 phosphorylation in various oncogenic pathways (reviewed in [15]). However, active GSK3β does not suppress the development and progression of tumors by disrupting the above-mentioned pro-oncogenic pathways and EMT, nor does the inhibition of GSK3β directly enhance tumorigenicity. Lithium is an ATP non-competitive and non-specific GSK3 inhibitor prescribed for bipolar disorder [16]. Long-term lithium treatment is associated with reduced kidney function and thyroid and parathyroid activity [17]. However, lithium treatment did not increase the incidence or mortality of cancer in large cohorts of patients with bipolar disorder compared to matched controls without lithium treatment [18,19]. Notably, in one of the latter control cohorts an increased risk of respiratory, gastrointestinal and endocrine cancers was observed compared to lithium-treated patients [18]. A recent systems biology study performed pathway and network enrichment analysis to explore the role of lithium in multiple cancer types and cancer-associated pathways, indicating the possible negative influence of lithium on the incidence of cancer and its therapeutic effects against cancer [20], as also discussed in the next section. These clinical observations on lithium treatment suggest that ATP-non-competitive inhibition of GSK3β is unlikely to cause tumor development and progression.

In contrast to the notion of GSK3β as a tumor suppressor, a growing number of studies over the past 15 years by our group and many others have instead shown that aberrant expression and activity of GSK3β facilitates the progression of various cancer types (reviewed in [21,22,23]). Pro-oncogenic activity for GSK3β is supported by the observation that deregulated GSK3β sustains tumor cell survival, proliferation and invasion by abrogating distinct tumor suppressor pathways and by enhancing cell immortality as well as the machinery for cell motility and migration. GSK3β also renders cancer cells resistant to chemotherapy, ionizing radiation and some molecular targeted agents [15]. In summary, accumulating evidence defines GSK3β as a potential therapeutic target in cancer [15,21,22,23], thus encouraging the development of GSK3β inhibitors for cancer treatment [24,25].

## 3. Tumor-Promoting Roles of GSK3β in Various Cancer Types

To date, the tumor-promoting functions of deregulated GSK3β have been reported in 25 cancer types from various organs and tissues across the body (Table 1) [26,27,28,29,30,31,32,33,34,35,36,37,38,39,40,41,42,43,44,45,46,47,48,49,50,51,52,53,54,55,56,57,58,59,60,61,62,63,64,65,66,67,68,69,70,71,72,73,74,75,76,77,78,79,80,81,82,83,84,85,86,87,88,89,90,91,92,93,94,95,96,97,98,99,100,101,102,103,104,105,106,107,108,109,110,111,112,113,114,115,116,117,118,119,120,121,122,123,124,125,126,127,128,129,130,131,132,133,134,135,136,137,138,139,140,141,142,143,144,145,146,147,148,149,150,151,152,153,154,155,156,157,158,159,160]. The majority of these studies have also demonstrated therapeutic effects against the respective cancer types using various pharmacological GSK3β inhibitors including lithium, natural products and medicines with GSK3β-inhibiting activity, and GSK3β-specific short interfering (si) or short hairpin (sh)RNA that are highly selective for GSK3β inhibition. The most extensively investigated cancer types to date are colon and rectum [161], pancreas [162,163,164,165] and prostate [166], as well as glioblastoma [167,168,169] and leukemia [170,171,172]. Compared to GSK3β, only a few studies have investigated the tumor-promoting role of GSK3α, another isoform of the GSK3 family, in pancreatic cancer and acute myeloid leukemia [173,174,175].

Despite the concerns about putative tumor suppressor functions for GSK3β as outlined in the previous section, early phase clinical trials for solid cancer and leukemia have tested synthetic pharmacological GSK3β inhibitors, lithium and approved medicines with the ability to inhibit GSK3β (Table 2). Although these studies are still ongoing [176], some preliminary results have been published [119,177,178].

## 4. Aberrant GSK3β and the Hallmark Properties of Cancer

**Figure 1:**
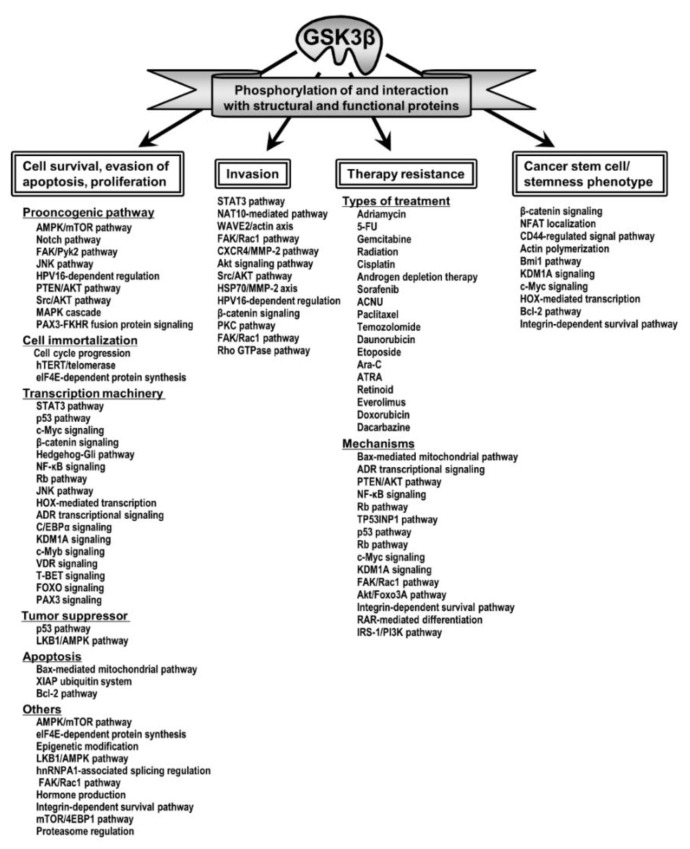
The tumor-promoting roles of GSK3β and the underlying mechanisms and pathways reported in the literature.

Thorough characterization of the underlying mechanistic basis for a novel therapy in the investigational phase is critical before it can proceed to clinical evaluation. Here we describe the pathological roles of deregulated GSK3β within the major hallmark properties of cancer [179], including tumor cell survival and proliferation, invasion, resistance to therapy and the tumor “stemness” phenotype (Table 1, Figure 1).

### 4.1. GSK3β and Tumor Cell Survival, Evasion of Apoptosis and Proliferation

The most pronounced and common hallmark property of cancer is persistent tumor cell survival with evasion of apoptosis and proliferation [179]. As shown in Table 1, GSK3β sustains tumor cell survival in many cancer types by exploiting various pro-survival pathways mediated by nuclear factor (NF)κ-B [48,52,53,54,55,63,78,94,95,98,107,128,129,151,153], Hh/Gli [43], mammalian target of rapamycin (mTOR) [97,140] and signal transducers and activators of transcription (STAT)3 [27,68]. Additionally, GSK3β helps tolerate apoptotic stimuli induced by the tumor necrosis factor-related apoptosis inducing ligand (TRAIL) receptor-dependent synthetic lethal system [36,57,61,71,74,83,107]. GSK3β can also perturb the p53-mediated tumor suppressor pathway [34,35,40,103,127,158] and Rb-mediated cell cycle regulatory machinery [29,62,109]. Sustained activity of human telomerase reverse transcriptase (hTERT) and telomerase in response to aberrant GSK3β contributes to the immortalization of tumor cells from the colon and rectum, pancreas, liver, lung, urinary bladder, ovary and uterine cervix [29,38]. Cell proliferation pathways mediated by c-Myc, cyclin D1 and STAT3 can promote unrestrained GSK3β-dependent tumor cell proliferation [51,52,53,68,97,101,102,107,109,113,122,126,157]. 

The dual functions of β-catenin consist of cell-to-cell adhesion and transcriptional co-activation of the T-cell factor (Tcf)/lymphoid enhancer factor (Lef) transcription factor. These functions depend on its subcellular localization in the cell membrane and nucleus and are responsible for tumor-suppressive and tumor-promoting roles, respectively, in several cancer types including colorectal cancer [180,181]. Paradoxically, the induction of Wnt/β-catenin signaling through inhibition of GSK3β has been shown to suppress tumor cell survival and proliferation in osteosarcoma and rhabdomyosarcoma [152,157], pancreatic cancer and non-small cell lung cancer (NSCLC) [51,70,77]. This indirectly supports the notion that β-catenin acts as a tumor suppressor in these tumors (reviewed in [15]). It has been reported that inhibition of GSK3β in pancreatic cancer and NSCLC stabilizes β-catenin and thereby induces tumor cell death via transactivation of pro-apoptotic c-Myc [51]. Another study reported that upregulated β-catenin signaling does not affect the survival of pancreatic cancer cells during inhibition of GSK3β [70]. This suggests that a specific level of β-catenin signaling activity is required for tumor formation since excessive accumulation (activation) of β-catenin in normal and cancer cells leads to apoptosis [182,183]. It was also reported that β-catenin levels vary in different lung cancer cell lines undergoing knockdown of GSK3β. This indicates that GSK3β may function independently of the β-catenin pathway in lung cancer, consistent with previous reports on colorectal, stomach, pancreatic and liver cancers [33,184,185,186]. In embryonal rhabdomyosarcoma, inhibition of GSK3β activates the canonical Wnt pathway by stabilizing β-catenin, leading to reduced tumor proliferation and differentiation of tumor stem-like cells and a reduction in their self-renewal capacity [156]. These results are consistent with a study showing the Wnt/β-catenin pathway is essential for the transition from stem cell self-renewal to myogenic differentiation during muscle regeneration [187]. The putative tumor suppressor role of this pathway in osteosarcoma has yet to be investigated and is discussed further in Section 5.5.

Mitosis is a direct driving force for cancer cell propagation and has therefore long been recognized as a therapeutic target in cancer [188,189,190]. Previously, our group and others showed that GSK3β inhibition in colorectal, pancreatic and breast cancer cells induced mitotic catastrophe by disrupting biodynamic processes during the formation of mitotic microstructures (centrosomes, spindle apparatus and chromosomes), ultimately resulting in apoptosis [47,50]. This observation points to a critical role for GSK3β in the mitotic process.

Elevated glycolysis is one of the hallmark metabolic properties of cancer cells and provides strong selective pressure for malignant evolution in most cancer types [191,192,193]. Intermediate metabolites in the glycolysis pathway fuel the synthesis of biomacromolecules such as nucleic acids and structural proteins required for mitosis [191,192]. A recent preliminary study by our group (Bolidong D. et al., unpublished) revealed that GSK3β phosphorylates and inactivates glycogen synthase in esophageal squamous cell carcinoma (ESCC), which is characterized biochemically by the depletion of intracellular glycogen [194]. This observation suggests that deregulated GSK3β may shift ESCC cell metabolism from glycogenesis to the glycolytic pathway, thus fueling cell proliferation. Another previous study showed that GSK3β increased protein synthesis, thereby enhancing cell proliferation in breast cancer through regulation of the eukaryotic translation initiation factor 4E (eIF4E)-binding protein 1 (4E-BP1) [80]. In summary, GSK3β contributes to tumor cell survival and proliferation by interacting with distinct pro-oncogenic pathways, the cell cycle pathway, the mitotic process and probably also aberrant glycolysis.

### 4.2. GSK3β and Tumor Invasion

Tumor invasion of host tissues and organs generates the distinctive tumor microenvironment that is critical for metastasis, thus remaining a major challenge in the treatment of cancer [195,196]. The most noticeable cellular phenotype responsible for tumor invasion and metastasis is epithelial–mesenchymal transition (EMT). EMT is defined as the acquisition of mesenchymal phenotypes, both biological and morphological, by tumor cells of epithelial origin [197,198,199], although some controversies still exist [200]. An earlier study demonstrated that GSK3β inhibits transcription of snail, a repressor of E-cadherin, thus inducing EMT in normal breast epithelial cells [201]. This result suggests that GSK3β compromises the ability to invade by targeting the EMT mediator. However, no studies to date have shown that GSK3β inhibits EMT in tumor cells and attenuates their ability to invade. On the other hand, there is evidence that GSK3β participates in cytoskeletal organization, cell polarity, motility and migration during wound healing [202]. These same cellular events are also shared by tumor invasion.

Previous studies reported that lithium and GSK3-inhibiting indirubins decreased the migration and invasion of glioblastoma cells [108,112], suggesting a putative role for GSK3β in tumor invasion. Subsequently, we demonstrated that pharmacological GSK3β-specific inhibitors reduced the migration and invasion of pancreatic cancer cells [62] and glioblastoma cells [116], both of which are highly invasive tumor types [203,204]. Inhibition of GSK3β was observed to suppress the formation of lamellipodia and invadopodia, which are the horizontal and vertical cell margin microstructures responsible for cell migration and stromal degradation [205,206]. These morphological changes in tumor cells induced by GSK3β inhibition coincided with the disruption of pathways that are mediated sequentially by focal adhesion kinase (FAK), guanine nucleotide exchange factors (GEFs), Rac1 and c-Jun N-terminal kinase (JNK) (reviewed in [15]). Other studies have also demonstrated the pro-invasive nature of GSK3β in colorectal, pancreatic and breast cancer cells via the modulation of cytoskeletal microstructures and cytokine-mediated extracellular matrix degradation [44,64,69]. Together, these studies provide evidence that GSK3β enhances the process of tumor invasion and probably also that of metastatic spread.

### 4.3. GSK3β and Therapy Resistance

Resistance to therapy is an intractable biological characteristic of cancer and remains a major barrier to the success of current treatments with chemotherapeutics and radiation, as well as more recent molecular-targeted and immune-modulating agents [207]. Key biological events and determinants of resistance to cancer therapy include the ability of tumor cells to survive therapeutic insults, tumor heterogeneity, physical barriers to therapeutics due to intermingled stromal tissues, inflammatory and immune reactions in the tumor microenvironment, the presence of mutations in driver genes (e.g., K-*ras*) with no known inhibitors, and the consequences of therapeutic pressures [208]. In addition, a causal and pernicious interconnection between cancer invasion and therapy resistance has emerged which favors treatment failure [209]. In light of this, we previously reviewed the pivotal role of GSK3β as a hub that tightly connects the pathways and cellular events responsible for tumor invasion and resistance to therapy. We also documented how tumor types that acquire pro-invasive capacity as they evade therapeutic insults are also susceptible to experimental therapy that targets GSK3β [15].

A combination of multiple agents having different targets and mechanisms of action is frequently used to treat many diseases in order to optimize therapeutic efficacy, minimize adverse effects and prevent the development of therapy resistance. For the treatment of refractory cancers, molecular-targeted therapy is typically prescribed in combination with conventional chemotherapeutics and/or radiation therapy and with other targeted agents [210,211]. As shown in Table 1 and Figure 1, several studies have reported that inhibition of GSK3β enhances the efficacy of chemotherapeutic agents and radiation in various cancer types. Conversely, this indicates that GSK3β renders tumor cells insensitive to cancer therapy. Importantly, these therapy resistant tumor types share the same pathways with their capacity of invasion, suggesting that GSK3β forms a pernicious cycle between tumor invasion and resistance to therapy in the refractory cancer types [15].

### 4.4. GSK3β, Cancer Stem Cells and the “Stemness” Phenotype

Cancer initiating or stem-like cells (CSCs) are assumed to be at the origin of heterogeneous tumor cell populations in a broad spectrum of hematologic and solid malignancies [212]. Based on the theory of clonal evolution of tumorigenesis and on the normal stem cell (SC) concept [213], CSCs are defined conceptually as tumor cells with self-renewal capacity and pluripotent capabilities responsible for proliferation, invasion and metastasis, resistance to therapy and tumor relapse after surgery and adjuvant therapies [212,214]. Therefore, CSCs and related “stemness” phenotypes are potential targets in cancer treatment, albeit currently less feasible than other well-known targets [215]. Over the past several years, various compounds aimed at CSCs or “stemness” phenotypes have been developed, with some undergoing testing in clinical trials [216,217]. However, neither the identification nor the therapeutic targeting of CSCs has been as straightforward as initially hoped [212].

As discussed above, GSK3β participates in tumor cell survival, proliferation, invasion and therapy resistance. Considering the multiple roles played by CSCs in the biological hallmarks of cancer, a working hypothesis is that GSK3β is centrally involved in the underlying mechanism for sustaining CSC phenotypes. CSCs have been identified in glioblastoma and leukemia where they have undergone extensive studies [218,219]. As summarized in Table 1, an earlier study showed that GSK3β suppresses the differentiation of glioblastoma SCs in association with Bmi1, a polycomb group gene required for the self-renewal of neural stem cells [110]. Another study showed that GSK3β phosphorylates lysine-specific histone demethylase 1A (KDM1A), allowing stabilization by ubiquitin-specific peptidase (USP)22 and thereby repressing the transcription of *BMP2*, *CDKN1A* and *GATA6,* and ultimately resulting in the self-renewal of glioma SCs [117]. Recently, our group screened compound libraries and identified kenpaullone, a pharmacological GSK3β inhibitor that attenuates the survival of patient-derived glioblastoma SCs via the c-Myc-mediated pathway [122]. In leukemia, GSK3β maintains the mixed-lineage leukemia (MLL) SC transcriptional program mediated by homeobox (HOX). This follows the conditional association of cyclic (c)AMP response element binding protein (CREB) and its co-activators TOR complex (TORC) and CREB-binding protein (CBP) with homeodomain protein MEIS1 (Meis homeobox 1), a critical component of the MLL-subordinate program [132]. It was also reported that GSK3β inhibitors suppress Bcl2-mediated and α5/β1-integrin-dependent cell survival pathways, thereby eliminating primitive leukemia progenitor/stem cells [134,137,138]. Other studies have implicated different mechanisms for the effects of GSK3β inhibition on CSCs from colorectal, head and neck and prostate cancer [42,49,76,92].

In contrast to the role of GSK3β in CSCs, previous studies have indicated that GSK3β inhibition is essential for maintaining the “stemness” phenotype in embryonic and hematopoietic SCs. This is thought to be achieved through activation of the canonical Wnt/β-catenin and Hh signaling cascades and by regulating cytoskeletal rearrangement [220,221,222,223], consistent with the physiological roles of GSK3β in normal cell biology [3,4]. Such reverse roles for GSK3β between normal and neoplastic SCs (reviewed in [170,171,172]) may ensure the safety of CSC-targeted therapy using GSK3β inhibition. Future studies on the role of GSK3β in normal and cancer SCs should; therefore, be aimed at elucidating the biological mechanisms that underlie selective eradication of CSCs.

**Figure 2:**
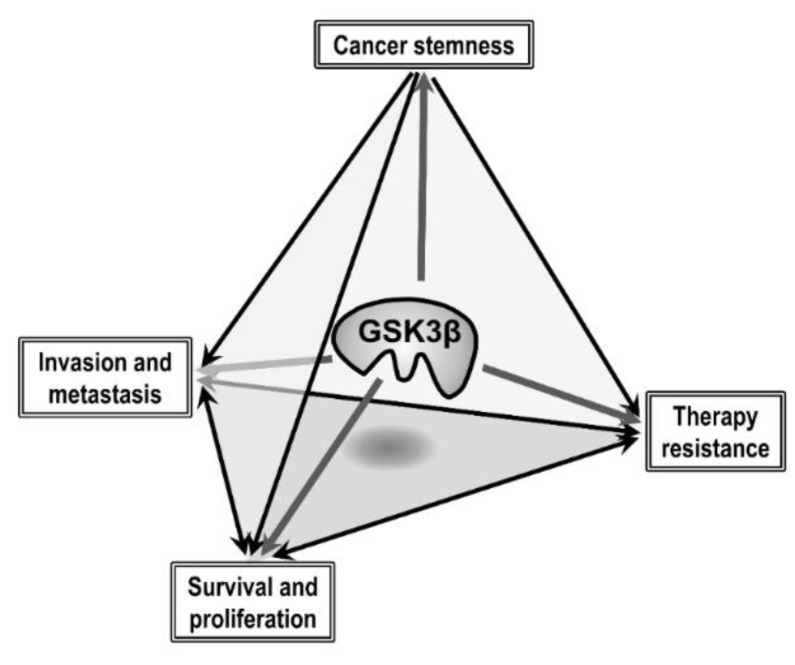
Schematic representation of the hypothesis that GSK3β is central to the interconnection between several of the biological hallmarks of cancer, including tumor cell survival and proliferation, invasion and metastasis, cancer stemness and resistance to therapy.

In summary, the evidence described in this section places GSK3β at the center of a trigonal intersection between the biological hallmarks of cancer, notably tumor cell survival and proliferation, invasion, resistance to therapy and CSC phenotype (Figure 2).

## 5. Protection of Normal Cells during Cancer Therapy by Targeting GSK3β

Targeting GSK3β for the treatment of diseases has raised concerns regarding the development and progression of cancer due to the promotion of proto-oncogenic pathways mediated by Wnt/β-catenin and Hh signaling [6,13,14]. Another concern is the overall safety of systemic GSK3β inhibition, as this could have undesirable consequences following the disruption of multiple signaling pathways. However, as previously reviewed by our group [15,21], it has yet to be demonstrated that GSK3β inhibition triggers neoplastic transformation or promotes any oncogenic process in normal cells. None of the studies on the tumor-promoting roles of GSK3β (Table 1) [26,27,28,29,30,31,32,33,34,35,36,37,38,39,40,41,42,43,44,45,46,47,48,49,50,51,52,53,54,55,56,57,58,59,60,61,62,63,64,65,66,67,68,69,70,71,72,73,74,75,76,77,78,79,80,81,82,83,84,85,86,87,88,89,90,91,92,93,94,95,96,97,98,99,100,101,102,103,104,105,106,107,108,109,110,111,112,113,114,115,116,117,118,119,120,121,122,123,124,125,126,127,128,129,130,131,132,133,134,135,136,137,138,139,140,141,142,143,144,145,146,147,148,149,150,151,152,153,154,155,156,157,158,159,160] showed any harmful effects of its inhibition on normal cells or vital organs in rodents. This is probably because GSK3β activity is finely controlled by a balanced, differential phosphorylation of its S9 and Y216 residues [3,4], unlike many cancer types where the activity is deregulated by an excess of Y216 over S9 phosphorylation. Such observations should dispel any concerns about the safety of GSK3β inhibition. They also highlight a major advantage of targeting GSK3β for cancer therapy in that it can spare normal cells and tissues from the toxic side effects seen with conventional cancer therapy.

### 5.1. GSK3β and Cancer Immunotherapy

Recent advances in immunotherapy hold considerable promise for more effective treatment of cancer [224]. Among the innate immune reactions against cancer, natural killer (NK) cells are capable of directly destroying cancer cells without being restricted by the major histocompatibility complex (MHC). This is due to their expression of a diverse array of germline-encoded activating and inhibitory receptors [225,226]. Clinical trials have tested different NK cell-based therapies for cancer, particularly for hematological malignancies, but their efficacy was not as high as anticipated [227]. Therefore, increasing the activity of NK cells against cancer is a promising avenue for the clinical application of immunotherapy [228]. Recently, two groups showed that GSK3β inhibition in normal peripheral NK cells enhances their cytotoxic effects against acute myeloid leukemia (AML) cells [143,144]. These effects were associated with increased AML-NK cell conjugates via upregulation of lymphocyte function-associated antigen (LFA) expression on NK cells and by inducing the expression of intercellular adhesion molecule-1 (ICAM-1) on AML cells [143]. Inhibition of GSK3β was shown to facilitate the maturation of peripheral NK cells via increased surface expression of CD57, thereby enhancing their cytotoxic activity [144]. Therefore, GSK3β inhibition in AML has the dual effects of directly suppressing tumor cell survival and proliferation, and of activating innate NK cells to destroy the tumor cells.

The function of CD8^+^ memory T-cells is adoptive anti-tumor immunity. Following GSK3β inhibition these cells dedifferentiate into pluripotent memory stem T-cells with anti-tumor capacity via activation of the Wnt/β-catenin pathway [229]. Consistent with this, a recent study showed that GSK3β inhibition increased the cytotoxic effect of CD8^+^ memory stem T-cells in gastric cancer through induction of effector T-cell-derived Fas-ligand [31]. Genetically engineered chimeric antigen receptor (CAR)-T cells have emerged as a new type of cancer immunotherapy and were recently approved for the treatment of leukemia and malignant lymphoma [230]. Similar to the effect on CD8^+^ memory T-cells, inhibition of GSK3β in mouse glioblastoma-specific CAR-T cells increased their survival, proliferation and memory phenotype generation, as well as enhancing their cytotoxic capacity [121]. These early results hold considerable promise for the targeting of GSK3β in T-cell-mediated anti-cancer immunotherapies.

Hematopoietic stem cell transplantation (HSCT) has long been the mainstay of curative therapy for hematological malignancies and most frequently for leukemia. However, its efficacy is diminished by graft versus host disease (GvHD). This immune complication occurs after both allogenic and autologous HSCT and is associated with considerable morbidity and mortality [231,232]. Immunosuppressive agents are used to prevent GvHD, but they increase the risk of disease relapse by inhibiting the graft versus leukemia effect. Thus, new treatments that prevent the relapse of leukemia are urgently required to address this serious concern. A previous study demonstrated that 3,6-bromoindirubin 3′-oxime (BIO), a GSK3β inhibitor, prevents lethal GvHD in a humanized xenograft in mice without affecting donor T-cell engraftment [233]. It also showed that BIO suppresses donor T-cell activity while reducing damage to bone marrow and liver by active donor T-cells. Subsequent studies showed that treatment with BIO preserves naïve T-cell phenotype by activating Wnt/β-catenin and c-myc signaling pathways in mice with reconstituted bone marrow, thereby promoting early engraftment of ex vivo-expanded hematopoietic stem cells [234,235]. These experimental studies suggest a potential role for GSK3β inhibition in the prevention of GvHD.

### 5.2. GSK3β and Cancer Therapy-Induced Hematotoxicity

Hematotoxicity is defined as the unfavorable effects of toxic substances or stimuli on the hematopoietic system including erythrocytes, leukocytes and platelets [236]. Various cancer therapy regimens with chemotherapeutic agents and radiation are frequently associated with hematotoxicity due to their induction of heavy oxidative stress in healthy cells [237,238]. Therapy-induced hematotoxicity mainly involves leukocytopenia, thrombocytopenia and to a lesser extent erythrocythemia (anemia). It is often a limiting factor in cancer therapy and is occasionally lethal [237]. Interventions using pharmacological agents with antioxidant properties have failed to prevent hematotoxicity [239]. As discussed in Section 4.4, previous studies showed that inhibition of GSK3β is a prerequisite for “stemness” in hematopoietic SCs [220,221,222,223]. An earlier study also showed that upon S9 phosphorylation mediated by phosphoinositide 3 kinase (PI3K) signaling, GSK3β becomes inactive in platelets that have been stimulated with hemo-coagulant factors such as collagen and thrombin [240]. Moreover, GSK3β inhibitors suppress the aggregation of platelets, suggesting that GSK3β negatively regulates platelet functions. It is, therefore, conceivable that GSK3β inhibitors could mitigate the hematotoxicity associated with chemotherapy and radiation.

### 5.3. GSK3β and Therapy-Induced Central and Peripheral Neuropathy

Chemotherapy-induced peripheral neuropathy (CIPN) is one of the most frequently encountered adverse events in cancer patients, particularly those treated with taxanes and platinum derivatives. Sensory symptoms for CIPN include pain, sensory loss, paresthesia and numbness, typically in the hands and feet. These symptoms often limit the dose of chemotherapeutic agents that can be used and persist after the completion of scheduled chemotherapy [241]. Based on the putative biological and molecular mechanisms underlying CIPN [242], randomized clinical trials have tested various pharmacological agents for the treatment of this disorder. Only a phase-III trial with duloxetine has so far shown any significant efficacy. Following the results of these clinical trials, the National Cancer Institute’s Symptom Management and Life Steering Committee has recognized CIPN as a priority area for translational research in cancer care (reviewed in [243,244]).

Since the pioneering study demonstrating that inhibition of GSK3β protects primary neurons of both the central and peripheral nervous systems [245], mounting evidence has confirmed the neuro-protective role of GSK3β inhibition [5,6,7]. Clinical trials have evaluated seed compounds for GSK3β inhibitors (e.g., tideglusib) in the treatment of Alzheimer’s disease and bipolar disorder (reviewed in [15]). A recent study showed that dual inhibition of GSK3β and CDK5 protects the cytoskeleton of neurons from neuroinflammatory-mediated degeneration, a common biological characteristic of neurodegenerative disorders [246]. Co-administration of pharmacological GSK3β inhibitors prevents apoptosis of neural precursor cells and peripheral neuropathy induced by camptothecin and paclitaxel without impairing their chemotherapeutic efficacy [247,248].

Cranial irradiation is essential for the treatment of patients with brain tumors including glioblastoma. However, long-term or persistent cognitive deficit with impaired learning and memory often occurs as a consequence of radiation-induced hippocampal damage [249,250]. Consistent with the neuroprotective effect of GSK3β inhibition described above, experimental studies showed that pretreatment with GSK3β inhibitors prevents radiation-induced neuronal apoptosis in the subgranular zone of the hippocampus in irradiated mice, consequently improving their cognitive functions. This effect is associated with the reversal of radiation-induced p53 stabilization and repair of DNA double-strand breaks [251,252]. In addition to intracranial radiation, prophylactic chemotherapy directed at the central nervous system (CNS) increases the survival of children with leukemia. However, late neurocognitive sequelae remain a serious concern with this treatment [253]. A recent study investigating adult survivors following CNS-directed chemotherapy with methotrexate for childhood leukemia identified phosphorylated tau (p-tau) in cerebrospinal fluid as a predictor of late neurocognitive sequelae [254]. This study suggests a possible involvement of GSK3β in the pathogenesis of neurocognitive sequelae, since tau is a well-known substrate of GSK3β for phosphorylation and stabilization [3,4,5]. Moreover, p-tau together with β-amyloid are recognized pathogenic substances in neurodegenerative diseases [6,7,8]. Consequently, inhibition of GSK3β is a promising strategy for the prevention and treatment of harmful side effects in the central and peripheral nervous system associated with cancer therapy.

### 5.4. GSK3β and Opioid-Induced Analgesic Tolerance and Withdrawal Syndrome

Management of common distress symptoms (e.g., pain, breathlessness, nausea and vomiting, fatigue) in advanced cancer patients is a vital part of palliative care. By improving the quality of life and preserving treatment compliance, the effective management of symptoms can also improve patient survival [255]. Opioids such as morphine are widely used to relieve pain in patients with advanced cancer and in those with intolerable pain due to diseases such as chronic pancreatitis. However, long-term treatment with opioids causes gradual progression of analgesic tolerance and the risk of withdrawal symptoms, thus limiting their use for adequate pain control in palliative care [256].

Previous investigations of opioid-induced cellular events indicate that long term treatment with morphine suppresses activity of the PI3K/Akt pathway, resulting in activation of GSK3β via reduced S9 phosphorylation [257,258]. Consistent with this, subsequent studies showed that co-administration of lithium or pharmacological GSK3β inhibitors (BIO, SB216763, SB415286) with morphine attenuated chronic, morphine-induced tail-flick tolerance and alleviated withdrawal behaviors in rats under experimental pain stimuli [259,260,261]. Together, these studies suggest the involvement of GSK3β in undesirable, opioid-induced clinical events. GSK3β could, therefore, be a potential target that would allow adequate control of cancer pain by opioids.

### 5.5. GSK3β and Normal Tissue Damage Associated with Surgery for Cancer

Surgery remains the mainstay of treatment for patients with solid malignant tumors. However, the resultant defects in normal tissue adjacent to the tumor can be a serious issue, particularly for patients with musculoskeletal tumors such as bone and soft tissue sarcomas [262,263]. Adjuvant chemotherapy and radiation, either alone or in combination, are often used together with surgery to optimize tumor resection and minimize the defect in adjacent normal tissues [262,263]. In addition to these two adjuvant therapies, clinical trials have also begun to evaluate molecular-targeted agents for bone and soft tissue sarcomas, but have so far failed to show any significant efficacy [264,265]. Therefore, the identification of new therapeutic targets has been a high priority for the treatment of these tumors [266,267,268].

Recently, our group and others reported a therapeutic effect of GSK3β inhibition against osteosarcoma [151,152,153,154], rhabdomyosarcoma [155,156], synovial sarcoma and fibrosarcoma [157]. These malignancies comprise the majority of sarcomas encountered in orthopedics for surgical removal. The therapeutic effect was associated with activation of the β-catenin signaling pathway in osteosarcoma [152] and in rhabdomyosarcoma [156], consistent with the observation that Wnt/β-catenin signaling is inactivated in these sarcomas [269,270]. A previous study also reported that undifferentiated sarcoma (or malignant fibrous histiocytoma, MFH) develops from mesenchymal stem cells (MSCs) via inactivation of the Wnt pathway [271], suggesting a pathogenic role for GSK3β in this tumor type. Accumulating evidence has shown the Wnt/β-catenin pathway plays a key role in bone formation and homeostasis by inducing osteoblastogenesis and osteoblast differentiation, and by impairing osteoclastogenesis [272,273,274,275,276]. Osteoclasts in the tumor microenvironment have been shown to facilitate the progression of osteosarcoma [266]. Furthermore, inhibition of GSK3β protects skeletal muscle cells from apoptosis, promotes their differentiation [277,278] and sustains the “stemness” and proliferation of MSCs [279,280]. Therefore, targeting of GSK3β in musculoskeletal tumors may have three advantages: direct therapeutic effect against the tumor, reduction of normal tissue defect caused by surgical removal of the tumor, and enhancement of adjacent normal tissue preservation.

Collectively, it can be deduced from the above review of the literature that GSK3β-targeted cancer treatment would appear to confer much greater therapeutic advantages compared to the hypothetical risk of tumorigenesis.

## 6. Future Perspectives on GSK3β in Cancer Treatment

Current topics in oncology research and cancer therapies focus mainly on the regulation and targeting of immune checkpoints, the interleukin (IL)17-mediated T helper (Th)17 cell immune reaction and mutant K-*ras*-driven oncogenic signaling in cancer. Here we discuss the potential involvement of GSK3β in these emerging therapeutic targets.

### 6.1. GSK3β and the Regulation of Immune Checkpoints in Cancer

Immunomodulation as a strategy for cancer treatment has attracted high levels of interest due to its potential for clinical translation. Therapeutic blockade of immune checkpoints involves the programmed death (PD)-1 and PD-ligand (PD-L)1 axis, as well as cytotoxic T-lymphocyte-associated protein (CTLA)-4 [281,282]. Briefly, the interaction between PD-L1 expressed on cancer cells and PD-1 produced by CD8^+^ T-cells allows the cancer cells to evade the T-cell-based anti-cancer immune system. CTLA-4 belongs to the CD28 immunoglobulin superfamily and is expressed at the surface of both CD4^+^/CD8^+^ T-cells and CD25^+^/forkhead box P (FOXP)3^+^ regulatory T-cells. CTLA-4 competes with CD28 for binding to its ligands CD80 and CD86 on antigen-presenting cells, thus blocking T-cell immunity against cancer cells. Therapeutic antibodies against PD-1, PD-L1 and CTLA-4 have been evaluated in clinical trials of cancer treatment and several have been approved for the treatment of malignant melanoma and lung cancers. Gastrointestinal cancers also show response, in particular those with defective DNA mismatch-repair leading to microsatellite instability [282]. However, a large number of cancer patients undergoing treatment with these antibodies are unresponsive, highlighting the urgent need for accurate predictive biomarkers of treatment efficacy [283]. Treatment failure following immune checkpoint blockade is likely due to the evasion of cancer cells from the immune system, as well as innate and acquired therapy resistance [284,285]. While conventional chemotherapy and molecular-targeted therapy act mostly on cancer cells, immune checkpoint blockade can revitalize latent T-cell immunity resulting in “immune-related adverse events”. These events frequently involve the gastrointestinal tract, liver, endocrine glands and skin, and less frequently the CNS, respiratory, cardiovascular, hematopoietic and musculoskeletal systems (reviewed in [286,287]).

As described in Section 5.1, inhibition of GSK3β causes CD8^+^ memory T-cells to dedifferentiate into progenitor CD8^+^ memory stem T-cells that are capable of self-renewal and cytotoxic effects [229]. Recent studies found that inactivation of GSK3β decreases PD-1 expression by up-regulating the transcription factor Tbx21 (Tbet), thereby enhancing CD8^+^ cytotoxic T-cell responses [288,289]. Another study showed that inhibition of poly [ADP-ribose] polymerase (PARP)1 increased the expression of PD-L1 in breast cancer cells directly via activation of GSK3β [290], suggesting that GSK3β is required for PARP1-regulated PD-L1 expression. In addition to the role of GSK3β in immune checkpoints mediated by the PD-1/PD-L1 axis, it was reported that inhibition of GSK3β reverses the blockade of CD28 by CTLA-4 [291] required to rescue exhausted CD8^+^ T-cells [292]. Collectively, these studies suggest involvement of GSK3β in the regulation of immune checkpoints by the PD-1/PD-L1 axis and by CTLA-4 in the cancer immunoenvironment [293]. Further studies may provide new insights into the potential role of GSK3β in the immune checkpoint mechanisms in cancer. In particular, research should investigate whether inhibition of GSK3β can increase the efficacy of immune checkpoint blockade, combat therapy resistance and improve immune-related adverse events.

### 6.2. GSK3β and the Regulation of IL-17/Th17 Immunity

Interleukin (IL)-17 is a pleiotropic proinflammatory cytokine produced by CD4^+^ Th17-cells and by a variety of immune cells such as δγ T-cells. IL-17 signaling-mediated inflammation promotes cancer-elicited inflammation and angiogenesis, as well as protecting cancer cells from immune surveillance (reviewed in [294,295]). Pro-tumorigenic effects of the IL-17-mediated pathway have been reported in colorectal and pancreatic cancers, where tumor infiltration by Th17-cells has been correlated with tumor progression and worse patient outcomes [296,297,298]. These results suggest that agents (e.g., antibodies) which target IL-17 or its receptor, or which impair the generation of Th17-cells, may represent a new therapeutic option in these cancer types.

Th17 cells are generated through a STAT3-dependent mechanism and IL-17 is thought to promote tumorigenesis and the progression of colorectal and pancreatic cancers via activation of IL-6/STAT3 and NF-κB signaling pathways [299,300,301]. As described in Section 3 and Section 4, these cancer types have been extensively studied with regard to the tumor-promoting role of GSK3β (Table 1) [28,29,32,33,34,35,36,37,38,39,40,41,42,43,44,45,46,47,48,49,50,51,52,53,54,55,56,57,58,59,60,61,62,63,64,65,66,67,68,69]. It is also known that GSK3β enhances the STAT3-mediated pathway to facilitate tumor progression [30,302]. A previous study reported that GSK3β is a critical mediator of the differentiation of pathogenic Th17-cells via the IL6/STAT3 pathway in the mouse models of pulmonary bacterial infection and autoimmune encephalomyelitis (multiple sclerosis), respectively [303]. Taken together, these studies infer that GSK3β may positively regulate the tumor promoting function of IL-17/Th17 immunity, warranting further investigation.

### 6.3. GSK3β and the Therapeutic Targeting of K-Ras Mutant Tumors

Among the known cancer driver genes, gain-of-function mutation in the *ras* family of genes (K-, N- and H-*ras*) is very prevalent. K-*ras* mutations are detected in almost one third of all human cancers and are especially common in pancreatic, colorectal and lung cancers [304]. K-ras oncoprotein is a constitutively active GTPase and provokes a diverse array of oncogenic signaling pathways mediated by Raf/MAPK kinase (MEK)/extracellular signal-regulated kinase (ERK), PI3K/Akt, RalGDS/Ral, T-lymphoma invasion and metastasis-1 (TIAM1)/Rac and p190/Rho axes. Activation of these pathways eventually facilitates tumor cell survival, proliferation, invasion, distinct metabolic reprogramming and therapy resistance [305]. Patients with K-*ras*-mutant colorectal cancer show unfavorable prognosis due to lack of response to epidermal growth factor receptor (EGFR)-targeted agents. Unfortunately, direct targeting of the K-ras oncoprotein has proven to be extremely difficult and is widely considered to be “undruggable” despite several attempts having been made for drugging this oncoprotein [306,307,308,309,310].

Recently, two direct covalent inhibitors of mutant K-ras^G12C^ oncoprotein, AMG 510 and MRTX849, were evaluated in phase I first-in-human clinical trials. Objective responses to these inhibitors were observed in about half of patients with lung cancer harboring K-*ras*^G12C^ mutation [311,312]. However, similar to receptor-type tyrosine kinase (RTK) inhibitors [313,314], acquired resistance to the mutant K-ras^G12C^ inhibitors was found to develop in an experimental setting via bypassing their effects against tumor proliferation by production of oncoprotein that did not bind to the inhibitors [315,316]. A subsequent experimental study showed that co-administration of Src homology region 2 domain-containing phosphatase-2 (SHP2) abrogates the adaptive response of cancer cells to the mutant K-ras^G12C^ inhibitors. This resulted in suppression of the feedback reactivation of MAPK signaling, thereby inhibiting tumor proliferation [317,318]. Eventually however, this strategy leads to a spiral of drug development followed by the emergence of resistance, similar to the experience with RTK inhibitors [313,314].

A recent study has attempted to address the above dilemma of drug resistance. It showed that GSK3β is required for the survival and proliferation of human colorectal and pancreatic cancer cells that depend on mutant K-*ras* [51]. Stabilization of β-catenin and c-Myc proto-oncoproteins, which are substrates for phosphorylation by GSK3β, was paradoxically associated with anti-tumor activity following GSK3β inhibition in these tumors. Inhibition of GSK3β also suppressed the growth of primary and metastatic patient-derived xenografts from pancreatic cancer patients who harbored K-*ras* mutations (G12D, G12V, G12C) and were resistant to chemo- and radiation therapies [51]. As described in Section 3 and Section 4, the therapeutic efficacy of GSK3β inhibition is well established in colorectal, pancreatic and lung cancers regardless of their K-*ras* mutation status (Table 1) [28,29,32,33,34,35,36,37,38,39,40,41,42,43,44,45,46,47,48,49,50,51,52,53,54,55,56,57,58,59,60,61,62,63,64,65,66,67,68,69,77,78,79], even though these cancer types are characterized by very frequent K-*ras* mutations. Furthermore, recent studies have suggested the potential of immunotherapy and in particular of adoptive T-cell therapy for the efficient targeting of mutant K-ras [319,320]. As discussed in Section 5.1 and Section 6.1, GSK3β is likely a negative regulator of adoptive T-cell-mediated immunity. Therefore, it would be interesting in future studies to elucidate whether adoptive T-cell-based and GSK3β-targeted therapies can synergize to overcome the resistance of K-*ras* mutant cancers to therapeutic agents.

## 7. Conclusions

This review has presented current knowledge regarding the tumor-promoting roles of GSK3β and the therapeutic efficacy of its inhibition. In addition, we describe potentially beneficial effects of GSK3β inhibition for the host and for normal cells following damage caused by conventional cancer therapy and palliative care. We also discussed the potential roles for GSK3β in sustaining the immune checkpoint machinery and IL-17/Th17 immunity, as well as in therapeutic targeting of K-*ras* mutant cancers. Taken together, this information provides a strong rationale for the targeting of GSK3β in the quest to cure cancer.

## Figures and Tables

**Table 1:** Tumor-promoting property of GSK3β reported in various cancer types.

Role of GSK3β in Biological Characteristics of Cancer					Reference
	Cell survival, evasion of apoptosis, and proliferation	Invasion	Therapy resistance *^1^	Cancer stem cell/stemness phenotype	
Digestive system					
Esophageal cancer(ESCC)	cell cycle progression; STAT3 pathway				[26,27]
Stomach cancer	hTERT/telomerase	STAT3 pathway			[28,29,30,31]
Colorectal cancer	p53 pathway; Bax-mediated mitochondrial pathway; TRAIL receptor-dependent synthetic lethal system; c-Myc signaling; β-catenin signaling; hTERT/telomerase; Hedgehog-Gli pathway; FAK/Pyk2 pathway; cell cycle progression; NF-κB signaling	N-acetyltransferase 10-mediated pathway; WAVE2/actin axis	adriamycin; 5-FU; Bax-mediated mitochondrial pathway; p53 pathway	β-catenin signaling; NFAT localization	[28,29,32,33,34,35,36,37,38,39,40,41,42,43,44,45,46,47,48,49,50,51]
Pancreatic cancer	NF-κB signaling; hTERT/telomerase; XIAP ubiquitin system; TRAIL receptor-dependent synthetic lethal system; JNK pathway; Rb pathway; Notch pathway; TFEB signaling; STAT3 pathway; c-Myc signaling; β-catenin signaling; cell cycle progression	FAK/Rac1 pathway; CXCR4/MMP-2 axis; Akt signaling pathway	TP53INP1 pathway; Rb pathway		[28,29,51,52,53,54,55,56,57,58,59,60,61,62,63,64,65,66,67,68,69,70]
Liver cancer (HCC)	Rb pathway; hTERT/telomerase; TRAIL receptor-dependent synthetic lethal system; cell cycle progression; NF-κB signaling				[28,29,71,72,73,74]
Head and neck cancer					
HNSCC	TLR-induced cytokine signaling			CD44-regulated signaling pathway	[75,76]
Lung cancer					
NSCLC	hTERT/telomerase; β-catenin signaling; NF-κB signaling				[38,77,78,79]
Breast cancer	eIF4E-dependent protein synthesis; epigenetic modification; cell cycle progression; PTEN/AKT pathway		PTEN/AKT pathway		[47,80,81,82]
Prostate cancer	TRAIL receptor-dependent synthetic lethal system; androgen receptor transcriptional signaling; cell cycle progression; C/EBPα signaling; Src/AKT pathway; LKB1/AMPK pathway	Src/AKT pathway	androgen receptor transcriptional signaling	actin polymerization	[83,84,85,86,87,88,89,90,91,92,93]
Urinary system					
Renal cell carcinoma	NF-κB signaling; AMPK/mTOR pathway, cell cycle progression		NF-κB signaling		[94,95,96,97]
Bladder cancer	hTERT/telomerase; NF-κB signaling; cell cycle progression	HSP70/MMP-2 axis			[38,98,99,100]
Female genital system					
Ovarian cancer	cell cycle progression; hTERT/telomerase; p53 pathway				[38,101,102,103,104]
Endometrial cancer	cell cycle progression		p53 pathway		[105]
Cervical cancer	hTERT/telomerase; HPV16-dependent regulation	HPV16-dependent regulation			[38,106]
Central and peripheral nervous system					
Glioblastoma	TRAIL receptor-dependent synthetic lethal system; c-Myc signaling; NF-κB signaling; Bax-mediated mitochondrial pathway; cell cycle progression; hnRNPA1-associated splicing regulation; KDM1A signaling; FAK/Rac1 pathway	β-catenin signaling; PKC pathway; FAK/Rac1 pathway; Rho GTPase pathway	p53 pathway; Rb pathway; c-Myc signaling; KDM1A signaling; FAK/Rac1 pathway; NFAT/FasL signaling	Bmi1 pathway; KDM1A signaling; c-Myc signaling	[107,108,109,110,111,112,113,114,115,116,117,118,119,120,121,122,123]
Neuronal tumors	hormone production; cell cycle progression; Myc signaling; p53 pathway				[124,125,126,127]
Hematopoietic system					
Leukemia	NF-κB signaling; β-catenin signaling; cell cycle progression; HOX-mediated transcription; integrin-dependent survival pathway; Bcl-2 pathway; c-Myb signaling; cell cycle progression; mTOR/4EBP1 pathway; MAPK cascade; VDR signaling; T-BET signaling		NF-κB signaling; Akt/Foxo3A pathway; integrin-dependent survival pathway; RAR-mediated differentiation	HOX-mediated transcription; Bcl-2 pathway; integrin-dependent survival pathway	[128,129,130,131,132,133,134,135,136,137,138,139,140,141,142,143,144]
Myeloma	FOXO signaling				[145]
Endocrine and neuroendocrine system					
Thyroid cancer	cell cycle progression; hormone production				[146,147]
Neuroendocrine tumors	proteasome regulation; cell cycle progression		cell cycle progression; IRS-1/PI3K pathway		[148,149,150]
Bone and soft tissue					
Osteosarcoma	NF-κB signaling; β-catenin signaling		NF-κB signaling		[151,152,153,154]
Soft tissue sarcomas	PAX3-FKHR fusion protein signaling; β-catenin signaling; cell cycle progression				[155,156,157]
Melanoma	p53 pathway; PAX3 signaling; β-catenin signaling		β-catenin signaling		[158,159,160]

*^1^ Therapy types (anti-cancer agents, radiation) and underlying mechanism(s).

**Table 2:** Clinical trials of GSK3β inhibitors for treatment of cancer *.

GSK3β Inhibitor (Company)	Cancer Type	Trial ID and Phase	Combined Regimen	URL (Access Date: 15 May 2020)	Reference
LY2090314 (Eli Lilly)	Acute leukemia	NCT01214603 Phase II	none	https://clinicaltrials.gov/ct2/show/NCT01214603	[177]
	Metastatic pancreatic cancer	NCT01632306 Phase I/II	Gemcitabine, FOLFOX, or Gemcitabine + nab-paclitaxel	https://clinicaltrials.gov/ct2/show/NCT01632306	
	Advanced or metastatic solid cancer	NCT01287520 Phase I	Pemetrexed + carboplatin	https://clinicaltrials.gov/show/NCT01287520	[178]
Lithium carbonate	Localized prostate cancer	NCT02198859 Phase I	none	https://clinicaltrials.gov/ct2/show/record/NCT02198859	
9-ING-41	29 advanced cancer types	NCT03678883	chemotherapeutics	https://clinicaltrials.gov/ct2/show/NCT03678883	
CLOVA cocktail	Advanced pancreatic cancer	UMIN000005095 Phase I/II	Gemcitabine	https://upload.umin.ac.jp/cgi-open-bin/ctr/ctr.cgi?function=brows&action=brows&type=summary&recptno=R000006032&language=E	
	Recurrent glioblastoma	UMIN000005111 Phase I/II	Temozolomide	https://upload.umin.ac.jp/cgi-open-bin/ctr/ctr.cgi?function=brows&action=brows&type=summary&recptno=R000002506&language=E	[119]

* The latest information was recently reviewed [176].

## Abbreviations

4EBP1	eukaryotic translation initiation factor 4E-binding protein 1
5-FU	5-fluorouracil
ACNU	nimustine hydrochloride
AMP	adenosine monophosphate
AMPK	AMP-activated protein kinase
Ara-C	cytosine arabinoside
ARHGAP	Rho GTPase-activating protein
ATRA	all-trans retinoic acid
Bax	Bcl-2-assosicated X protein
Bcl-2	B-cell lymphoma 2
Bmi1	B cell-specific Moloney murine leukemia virus integration site 1
CDK	cyclin-dependent kinase
C/EBPα	CCAAT/enhancer binding protein α
CLOVA	combined cimetidine, lithium chloride, olanzapine and valproate regimen
c-Myb	avian myeloblastosis virus oncogene cellular homolog
c-Myc	cellular myelocytomatosis
CXCR4	C-X-C chemokine receptor type 4
eIF4E	eukaryotic initiation factor-4E
ESCC	esophageal SCC
FAK	focal adhesion kinase
FasL	Fas ligand
FKHR	Forkhead in rhabdomyosarcoma
FOLFOX	combined folate, 5-fluorouracil and oxaliplatin regimen
FOXO	Forkhead box O
Gli	glioma-associated oncogene homologue
GSK3β	glycogen synthase kinase 3β
GTP	guanine triphosphate
HCC	hepatocellular carcinoma
hnRNPA1	heterogeneous nuclear ribonucleoprotein A1
HNSCC	head and neck SCC
HOX	homeobox
HPV	human papillomavirus
HSP	heat shock protein
hTERT	human telomerase reverse transcriptase
IRS-1	insulin receptor substrate 1
JNK	c-jun N-terminal kinase
KDM1A	lysine-specific demethylase 1A
LKB1	liver kinase B1
MAPK	mitogen-activated protein kinase
Mdm	mouse double minute
MMP-2	matrix metalloproteinase-2
mTOR	mammalian target of rapamycin
NFAT	nuclear factor of activated T cells
NF-κB	nuclear factor kappa B
NSCLC	non-small-cell lung cancer
PAX3	paired box 3
PI3K	phosphoinositide 3-kinase
PKC	protein kinase C
PTEN	phosphatase and tensin homolog deleted from chromosome 10
Pyk2	proline-rich tyrosine kinase 2
Rac1	RAS-related C3 botulinus toxin substrate 1
RAR	retinoic acid receptor
Rb	retinoblastoma
Rho	Ras homologous
SCC	squamous cell carcinoma
Src	Rous sarcoma oncogene cellular homolog
STAT3	signal transducer and activator of transcription 3
T-BET	T-box protein expressed in T cells
TFEB	transcription factor EB
TLR	Toll-like receptor
TNF	tumor necrosis factor
TP53INP1	tumor protein p53-inducible nuclear protein 1
TRAIL	TNF-related apoptosis-inducing ligand
VDR	vitamin D receptor
WASP	Wiskott–Aldrich syndrome protein
WAVE2	WASP-family verprolin homologous protein 2
XIAP	X-linked inhibitor of apoptosis protein
